# Functional roles of the pre-sensor I insertion sequence in an AAA+ bacterial enhancer binding protein

**DOI:** 10.1111/j.1365-2958.2009.06744.x

**Published:** 2009-06-23

**Authors:** Patricia C Burrows, Jörg Schumacher, Samuel Amartey, Tamaswati Ghosh, Timothy A Burgis, Xiaodong Zhang, B Tracy Nixon, Martin Buck

**Affiliations:** 1 Department of Life Sciences, Division of Biology, Imperial College LondonLondon, SW7 2AZ, UK; 2 Department of Life Sciences, Division of Molecular Biosciences, Imperial College LondonLondon, SW7 2AZ, UK; 3 Center for Bioinformatics, Division of Molecular Biosciences, Faculty of Natural Sciences, Imperial College LondonLondon, SW7 2AZ, UK; 4 406 Frear South Building, The Pennsylvania State University, University ParkPA 16802, USA

## Abstract

Molecular machines belonging to the AAA+ superfamily of ATPases use NTP hydrolysis to remodel their versatile substrates. The presence of an insertion sequence defines the major phylogenetic pre-sensor I insertion (pre-SIi) AAA+ superclade. In the bacterial σ^54^-dependent enhancer binding protein phage shock protein F (PspF) the pre-SIi loop adopts different conformations depending on the nucleotide-bound state. Single amino acid substitutions within the dynamic pre-SIi loop of PspF drastically change the ATP hydrolysis parameters, indicating a structural link to the distant hydrolysis site. We used a site-specific protein–DNA proximity assay to measure the contribution of the pre-SIi loop in σ^54^-dependent transcription and demonstrate that the pre-SIi loop is a major structural feature mediating nucleotide state-dependent differential engagement with Eσ^54^. We suggest that much, if not all, of the action of the pre-SIi loop is mediated through the L1 loop and relies on a conserved molecular switch, identified in a crystal structure of one pre-SIi variant and in accordance with the high covariance between some pre-SIi residues and distinct residues outside the pre-SIi sequence.

## Introduction

The large AAA+ (ATPases *a*ssociated with various cellular *a*ctivities) protein family are responsible for a wide range of ATP-dependent molecular transformations that involve the disassembly and remodelling of protein and nucleic acid complexes (Thomsen and Berger, 2008). Characteristically, all AAA+ members have the ability to self-assemble into higher-order oligomeric structures, usually as hexamers arranged in a ring (Hanson and Whiteheart, 2005). They contain the highly conserved Walker A and Walker B motifs, involved in ATP binding and ATP hydrolysis, the sensor I sequence and the second region of homology that comprises at least one intersubunit catalytic R-finger (Zhang *et al*., 2002). Sequence insertions prior to the sensor I motif and the preceding α-helix are regarded as a synapomorphic trait that defines the phylogenetic pre-sensor I insertion (pre-SIi) β-hairpin superclade within the AAA+ superfamily (Iyer *et al*., 2004). AAA+ members belonging to this superclade include HslU, ClpX, Lon, MCM, RuvB, the Ltag helicase and bacterial enhancer binding proteins (bEBPs; Schumacher *et al*., 2006). Although these sequence insertions (typically 15–25 amino acids in length) are not highly conserved among different AAA+ members, they are generally located in, or at the edge of the inner pore defined by the common hexameric AAA+ ring assembly (Iyer *et al*., 2004).

In a wide range of bacteria specialized AAA+ proteins (bEBPs), function to activate highly regulated adaptive responses mediated by the enhancer-dependent σ^54^-containing RNA polymerase (Eσ^54^) (Studholme and Dixon, 2003; Wigneshweraraj *et al*., 2008). Upon binding and hydrolysis of ATP, bEBPs remodel the initial closed promoter complexes formed by Eσ^54^ to create transcriptionally proficient open complexes (Popham *et al*., 1989). The bEBPs therefore effectively couple ATP hydrolysis to open complex formation where the DNA template strand is loaded within the active site of RNAP (Sasse-Dwight and Gralla, 1988; Morett and Buck, 1989; Popham *et al*., 1989). The major binding target for bEBPs is the σ^54^ factor (Bordes *et al*., 2003). Biochemical and structural analyses of bEBPs PspF, ZraR and NtrC1 (Lee *et al*., 2003; Rappas *et al*., 2005; Sallai and Tucker, 2005) have demonstrated that a direct interaction with σ^54^ occurs via the bEBP-specific GAFTGA motif, located within a structural insertion in Helix 3, termed the L1 loop (Bordes *et al*., 2003; Chen *et al*., 2007). The L1 loop is flanked by the pre-SIi [formerly referred to as the L2 loop, *Escherichia coli* residues 131–139 in PspF (Rappas *et al*., 2005)], and co-ordinated action between these two loop structures has previously been suggested (Schumacher *et al*., 2007). Allosteric co-ordination of nucleotide-dependent dynamics between the heterogeneously occupied subunits in the hexameric assembly are important for transcription activation, suggesting that only a subset of subunits directly act on Eσ^54^ (Joly *et al*., 2006; Schumacher *et al*., 2008).

Nucleotide analogues are commonly used to study structural transitions associated with ATP-dependent reactions (Wittinghofer, 1997). The ATP ground state analogue ADP–BeF causes a pronounced rising of the L1 and pre-SIi loops in the bEBP NtrC1, seen using small and wide angle X-ray scattering (SAXS/WAXS) analysis (Chen *et al*., 2007), a result consistent with the local changes observed in the ATP-bound crystal structure of PspF_1−275_ (Rappas *et al*., 2006). Further, σ^54^ can form stable complexes with NtrC1 and PspF in the presence of ADP–BeF (Chen *et al*., 2007). Interestingly in SAXS/WAXS analysis, the ATP analogues AMP·PNP and ATPγS did not cause detectable movements in these loop structures, although AMP·PNP was observed to stabilize complexes formed between σ^54^ and the bEBPs NtrC1 or PspF. Taken together, these results suggest that ATP binding causes the L1 and pre-SIi loops to rise, thereby supporting an initial unstable interaction between the bEBP and σ^54^ (Chen *et al*., 2007). Experimentally, stable complex formation between the bEBP and (E)σ^54^ occurs in the presence of the non-hydrolysable ATP hydrolysis transition state analogue ADP–AlF, which is thought to reflect the conformation of the bEBP–Eσ^54^ complex near to, or at the point of, an energy coupling step required for reorganization of the Eσ^54^ closed complex (Chaney *et al*., 2001; Rappas *et al*., 2005). ADP–AlF presumably ‘freezes’ the local motions at the ATPase catalytic site, which are linked to other dynamic excursions including the presentation of the L1 and pre-SIi loops to the closed complex.

Here we report a detailed analysis of the function of the flexible pre-SIi loop of PspF_1−275_. Single amino acid substitutions of the pre-SIi residues (131–139) to alanine invariably impacted on the ATPase kinetics, indicating a strong structural coupling between the ATP hydrolysis site and the distant pre-SIi loop. Differences in the functionalities of the pre-SIi variants enabled three classes of variants to be identified: (i) L1 loop facing; responsible for co-ordinating the position of the L1 loop; (ii) the pre-SIi loop tip; essential for all σ^54^ interaction and remodelling activities; and (iii) a more variable N-terminal side, which likely serves as a structural scaffold and contains residues important for ATPase activity. Our results establish that specific regions of the pre-SIi loop are important for engaging and remodelling the Eσ^54^ closed complex in response to different stages of the ATP hydrolysis cycle. We suggest that much, if not all, of the action of the pre-SIi loop is mediated through the L1 loop and relies on a conserved molecular switch, identified in a crystal structure of one pre-SIi variant and in accordance with the high covariance between some pre-SIi residues and distinct residues outside the pre-SIi sequence.

## Results

**Fig. 1:**
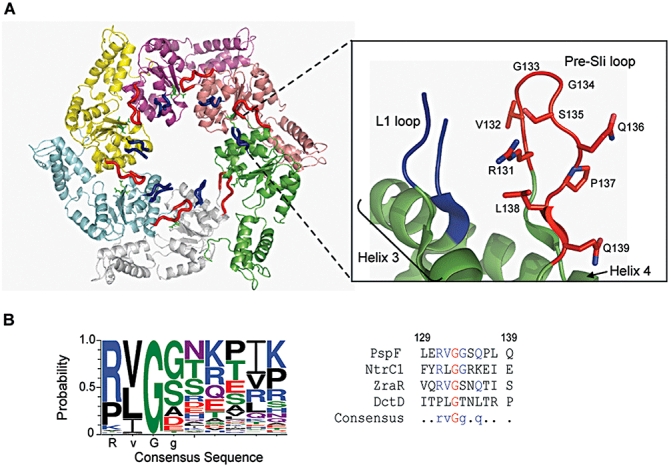
Location and sequence of the pre-SIi loop in PspF_1−275_. A. Crystal structure of the PspF_1−275_ hexamer with the location of the L1 and pre-SIi loops, Helix 3 and Helix 4 highlighted (PDB 2BJW). The residues that comprise the PspF pre-SIi are indicated. B. Left panel: The consensus pre-SIi insertion sequence obtained using the 289 annotated Pfam (00158) bEBP sequences. Alignment of these sequences suggests that conservation of the pre-SIi loop varies, with the RVGG motif being the most conserved sequence. Right panel: The pre-SIi sequences of structurally characterized bEBPs PspF_1−275_, NtrC1, ZraR and DctD.

Crystal structures of nucleotide-bound forms of PspF_1−275_, derived from soaking apo-PspF_1−275_ crystals with ADP, ATP and AMP·PNP revealed striking and distinct movements of the pre-SIi loop (Rappas *et al*., 2006). These structures suggest a structural coupling between the nucleotide binding site and the pre-SIi loop (Fig. 1A), which probably reflects nucleotide-dependent movements of the pre-SIi loop in solution. SAXS/WAXS data on the PspF homologue NtrC1 also provide evidence for different conformations of these loops in different nucleotide states (Chen *et al*., 2007). Figure 1B shows the pre-SIi loop consensus sequence obtained from 289 annotated Pfam (00158) bEBPs sequences. Alignment of these sequences (Fig. 1B) suggests that conservation of the pre-SIi loop (residues 131–139 in PspF) varies, with the RVGG motif (residues 131–134 in PspF, particularly the first glycine present in 99% of the available sequences) being the most conserved segment. To study the function of the pre-SIi loop in PspF, single amino acid substitutions to alanine were made across residues 131–139. For experimental simplicity, we used a structurally characterized form of PspF that is deleted for its DNA binding domain (AAA+ domain residues 1–275; PspF_1−275_), yet capable of activating transcription from σ^54^-dependent promoters both *in vivo* and *in vitro* from solution (Bordes *et al*., 2003). Notably, the sequence alignment (Fig. 1B) also indicated that the proline at position 137 in PspF (present in 21% of the available sequences) is replaced by a threonine residue in 14% of bEBPs. In order to ascertain the specific effect of the threonine at position 137, we also constructed a P137T variant.

With the exception of G134A, all clones directed the synthesis of soluble variant forms of PspF_1−275_. We infer that residue G134, which is located at the tip of the pre-SIi loop (see Fig. 1A), may be required for the structural stability of PspF_1−275_ because expression was below our detection limit.

### Transcription activation activities of some of the pre-SIi variants are compromised

**Fig. 2:**
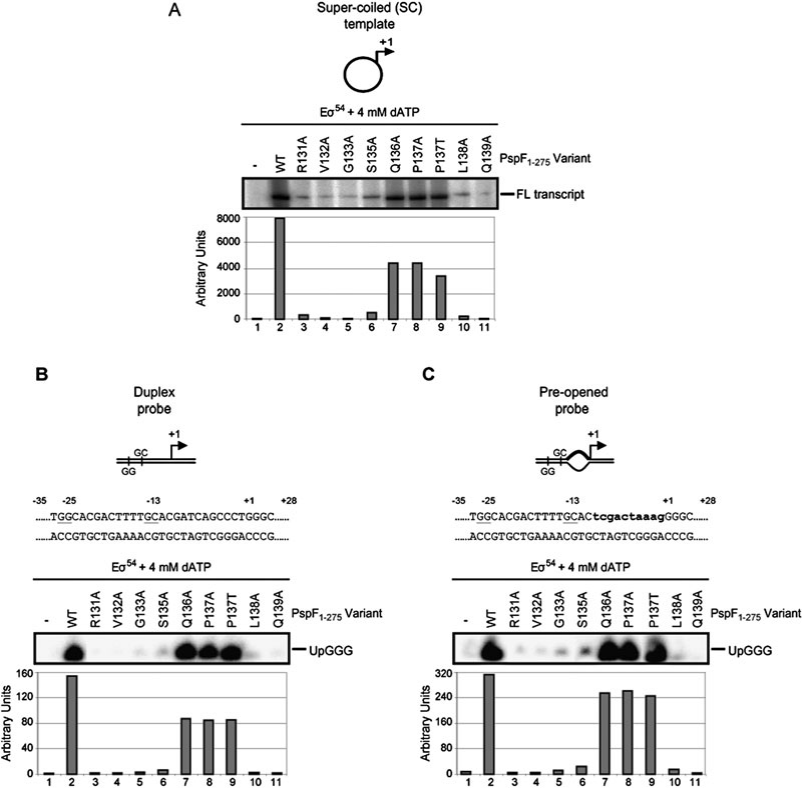
Specific pre-SIi variants demonstrate defects in transcription activation. A. Some pre-SIi variants fail to support Eσ^54^ transcription from the super-coiled (SC) *Sinorhizobium meliloti nifH* promoter (10 min activation time). The full-length (FL) transcript is as indicated. The Q136A and P137A/T variants demonstrate significant levels of transcription activity. B. Top: Schematic and nucleotide sequence of the *S. meliloti nifH* duplex promoter probe with the consensus promoter elements GG (positions −26 and −25) and GC (positions −14 and −13) underlined. Bottom: Abortive transcription gel showing the pre-SIi variants ability to support Eσ^54^ open complex formation on the linear duplex probe. The abortive transcript UpGGG is indicated. The relative numbers of complexes formed on the duplex probe in the presence of the pre-SIi variants are indicated in the graph below the gel. C. As in B but using the pre-opened promoter probe. The lowercase letters in bold type-face indicate the non-complementary residues in the pre-opened promoter probe.

The ability of the pre-SIi variants to support transcription activation was assessed using full-length transcription assays on the super-coiled *Sinorhizobium meliloti nifH* promoter. As shown in Fig. 2A, only two residues (Q136 and P137, lanes 7–9) in the pre-SIi loop were tolerant to alanine (or threonine) substitution, and supported near wild-type (WT, lane 2) levels of full-length transcription. These two residues are located farthest away from the L1 loop (see Fig. 1A) and do not appear to undergo any conformational or rotational changes in the presence of different nucleotides (Rappas *et al*., 2006). As these residues are not highly conserved among bEBPs (Fig. 1B), we suggest that Q136 and P137 may have little effect on the remodelling activities of PspF_1−275_ in particular the positioning or stabilization of the σ^54^ interacting L1 loop. We note that the S135A variant (lane 6) shows significantly reduced activity (compared with lane 2) as do the R131A (lane 3) and L138A (lane 10) variants, but the others are essentially unable to activate transcription.

Using abortive transcription assays (to measure heparin-resistant open complex formation; Wigneshweraraj *et al*., 2005) with two linear *nifH* promoter probes, a fully double-stranded (duplex) probe (Fig. 2B) and a pre-opened probe (Fig. 2C; mismatched between positions −10 and −1, thereby mimicking the DNA conformation within the Eσ^54^ open complex) (Wedel and Kustu, 1995; Cannon *et al*., 1999), we addressed whether pre-opening the DNA could rescue the transcription activation activity of any of the pre-SIi loop variants. Similar to the full-length transcription assays, only the pre-SIi variants Q136A and P137A/T were able to support open complex formation (Fig. 2B and C, lanes 7–9) at levels comparable to PspF_1−275_WT (lane 2), even in the presence of pre-opened DNA (Fig. 2C). We also note that the activity of the PspF_1−275_S135A variant still remained significantly lower than PspF_1−275_WT (Fig. 2B, lane 6). The remainder of the pre-SIi loop variants demonstrated a significantly reduced ability to support Eσ^54^ open complex formation. As pre-opening the DNA had little effect on the transcription activation activities of the majority of pre-SIi variants, we suggest that these variants have defects in steps that precede DNA melting. Clearly the pre-SIi loop is important for transcription activation with some residues making greater contributions than others.

### The transcription activation defective pre-SIi variants fail to stably interact with σ^54^

To assess the contribution of the pre-SIi loop in supporting σ^54^ binding and hence the Eσ^54^ transcription activation activities (of the L1 loop) we used a site-specific protein–DNA cross-linking method to study protein–DNA relationship changes for σ^54^–DNA, β/β′–DNA and PspF_1−275_–DNA interactions. The photo-reactive DNA templates were constructed by strategically placing the cross-linking reagent *p*-azidophenacyl bromide (APAB; Sigma), between positions −1 and +1 (−1/+1) in the context of the duplex, pre-opened and mismatch (at positions −12 and −11, mimicking the DNA conformation of the closed complex) promoter probes. The −1/+1 site was chosen based on previous observations (Burrows *et al*., 2009) that: (i) only σ^54^–DNA interactions are detected within the closed complex; (ii) in the ADP–AlF (intermediate) complex σ^54^–DNA and PspF_1−275_–DNA cross-linked species are detected; and (iii) only within the open complex are β/β′–DNA interactions observed corresponding to the loading of DNA within the active site of RNAP (Burrows *et al*., 2008; 2009).

**Fig. 3:**
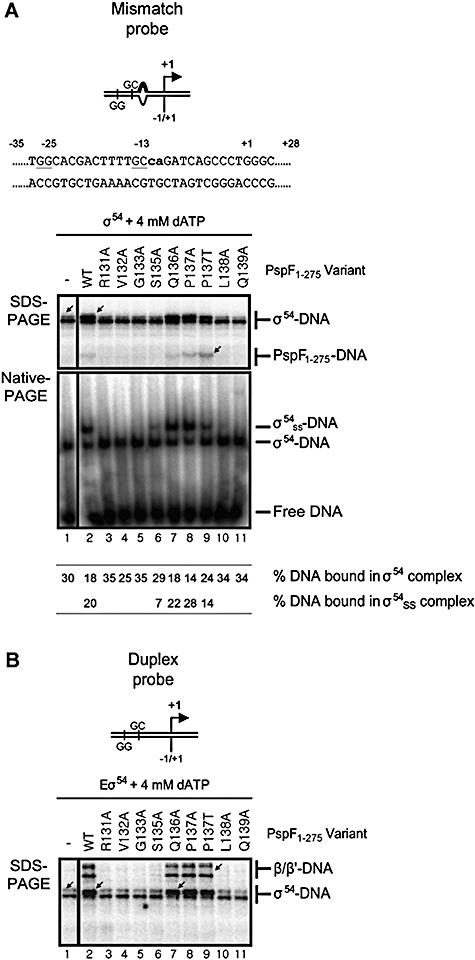
The pre-SIi variants defective for open complex formation fail to interact with σ^54^. A. Top: Schematic and nucleotide sequence of the *S. meliloti nifH* mismatch promoter probe, the lowercase letters in bold type-face indicate non-complementary residues in the mismatch promoter probe. Bottom: SDS-PAGE gel showing the cross-linking profiles of σ^54^–DNA complexes formed on the mismatch promoter probe in the presence of 4 mM dATP and either PspF_1−275_WT or variants. The migration positions of the cross-linked σ^54^–DNA and PspF_1−275_–DNA species are indicated. Native-PAGE gel illustrating supershift complexes (σ^54^ss–DNA) are formed in the presence of PspF_1−275_WT (lane 2) and the S135A (lane 6), Q136A (lane 7) and P137A/T (lanes 8 and 9) variants. The migration positions of the supershift (σ^54^ss–DNA) and binary σ^54^–DNA (σ^54^–DNA) complexes, free DNA and percentage DNA bound in each complex is indicated. B. SDS-PAGE gel as in A but in the presence of core RNAP. The migration positions of the cross-linked β/β′–DNA and σ^54^–DNA species are indicated. Cross-linked β/β′–DNA species are only observed with the transcription competent PspF_1−275_ variants (WT, Q136A and P137A/T).

Initially, we analysed a binary σ^54^–promoter complex formed on the mismatch promoter probe in the presence of PspF_1−275_ (WT and pre-SIi variants) and dATP. Previously we have shown that PspF_1−275_WT and dATP can remodel this binary σ^54^–DNA complex to a ‘supershift’ complex (Fig. 3A labelled σ^54^ss–DNA; Cannon *et al*., 2000; 2001). Using this assay, we can establish whether the defects observed in open complex formation by the pre-SIi variants (Fig. 2) are due to an inability to efficiently interact with σ^54^. In line with the transcription activation data (Fig. 2) only the Q136A, P137A/T and (to a lesser extent) S135A variants supported σ^54^ supershift complex formation (Fig. 3A, lanes 6–9); however, the remainder of the pre-SIi variants failed to support σ^54^ss–DNA complex formation (Fig. 3A, lanes 3–5 and 10–11). When these reactions were analysed by cross-linking we observed that where supershift complexes were present, the profile of the cross-linked σ^54^–DNA species was altered from a single band (Fig. 3A, SDS-PAGE; lane 1 arrowed) to a double band (Fig. 3A, lanes 2 and 7–9, lane 2 arrowed), suggesting that the organization of σ^54^ is changed in the σ^54^ss–DNA complex. However, no change in the σ^54^–DNA profile was observed in reactions containing S135A (Fig. 3A, lane 6), where a weak σ^54^ss–DNA complex was observed, or the other pre-SIi variants (131–133 and 138–139; lanes 3–6 and 10–11) where no σ^54^ss–DNA complexes were detected. We also note a weak cross-linked PspF_1−275_–DNA species was detected in the supershift reactions, particularly with the P137T variant (Fig. 3A, SDS-PAGE; lanes 2 and 7–9, lane 9 arrowed).

In the presence of core RNAP, the Q136A and P137A/T variants (Fig. 3B, lanes 7–9) supported a change in the σ^54^–DNA interactions (from a single band; lane 1 arrowed, to a double band, lane 7 arrowed) and formation of β/β′–DNA cross-links (lane 9 arrowed) comparable to PspF_1−275_WT. Together these data confirm that the transcription activation defect associated with some pre-SIi variants is due to their inability to productively interact with σ^54^ in the σ^54^–DNA complex or in the Eσ^54^ closed complex in the presence of dATP. Hence, defects in the pre-SIi variants occur at the level of the σ^54^ isomerization event, prior to the loading of DNA within the active site of RNAP.

### Pre-SIi variants impact on the ATP hydrolysis active site

Eσ^54^ open complex formation requires the coupling of energy derived from bEBP-dependent ATP hydrolysis to remodelling of the Eσ^54^ closed complex. Effective energy coupling relies on co-ordinated ATP binding and hydrolysis between different subunits of the hexameric PspF_1−275_ AAA+ ring assembly (Joly *et al*., 2007; Schumacher *et al*., 2008). Failure of the pre-SIi variants to activate transcription could therefore be a consequence of a failure to bind or hydrolyse ATP and/or to efficiently couple hydrolysis to restructuring of the Eσ^54^ closed complex.

To ascertain whether this was the case we measured the ATP binding activities of the pre-SIi variants using non-equilibrium UV cross-linking of [α-^32^P]-ATP (Schumacher *et al*., 2004; 2007). Results (Fig. S1) demonstrate that the PspF_1−275_ pre-SIi variants bind ATP with similar affinity as PspF_1−275_WT, indicating that the pre-SIi loop does not contribute to the nucleotide binding activities of PspF. In contrast, the pre-SIi variants appear to have a striking effect on the steady-state ATP hydrolysis rates (Table 1). We note however, there is no clear correlation between low ATP hydrolysis rates and defects in transcription activation activity, because the Q136A and T137A variants are similarly active in the transcription assays despite a more than 80% reduction in the ATPase activity of Q136A. The decrease in *V*_max_ observed for all the pre-SIi variants, except P137, indicates a finely tuned coupling between the structure of the pre-SIi loop and the distant ATP active site. These observations functionally distinguish the pre-SIi loop from the adjacent L1 loop, where alanine substitutions do not negatively impact on PspF_1−275_ ATPase activity (Rappas *et al*., 2005).

**Table 1:** ATP hydrolysis parameters of the PspF_1−275_ pre-SIi proteins.

PspF_1−275_ variant	*V*_max_ (min^−1^)	Standard error	*K*_m_ (μM)	Standard error
Wild type	30.57	3.46	178	61
R131A	< 0.1	ND	ND	ND
V132A	0.41	0.04	10	5
G133A	2.72	0.75	91	50
S135A	< 0.1	ND	ND	ND
Q136A	3.98	0.39	8	2
P137A	26.72	4.29	300	130
P137T	47.49	4.30	140	61
L138A	2.50	0.66	160	60
Q139A	< 0.1	ND	ND	ND

ND, not determined.

**Fig. 4:**
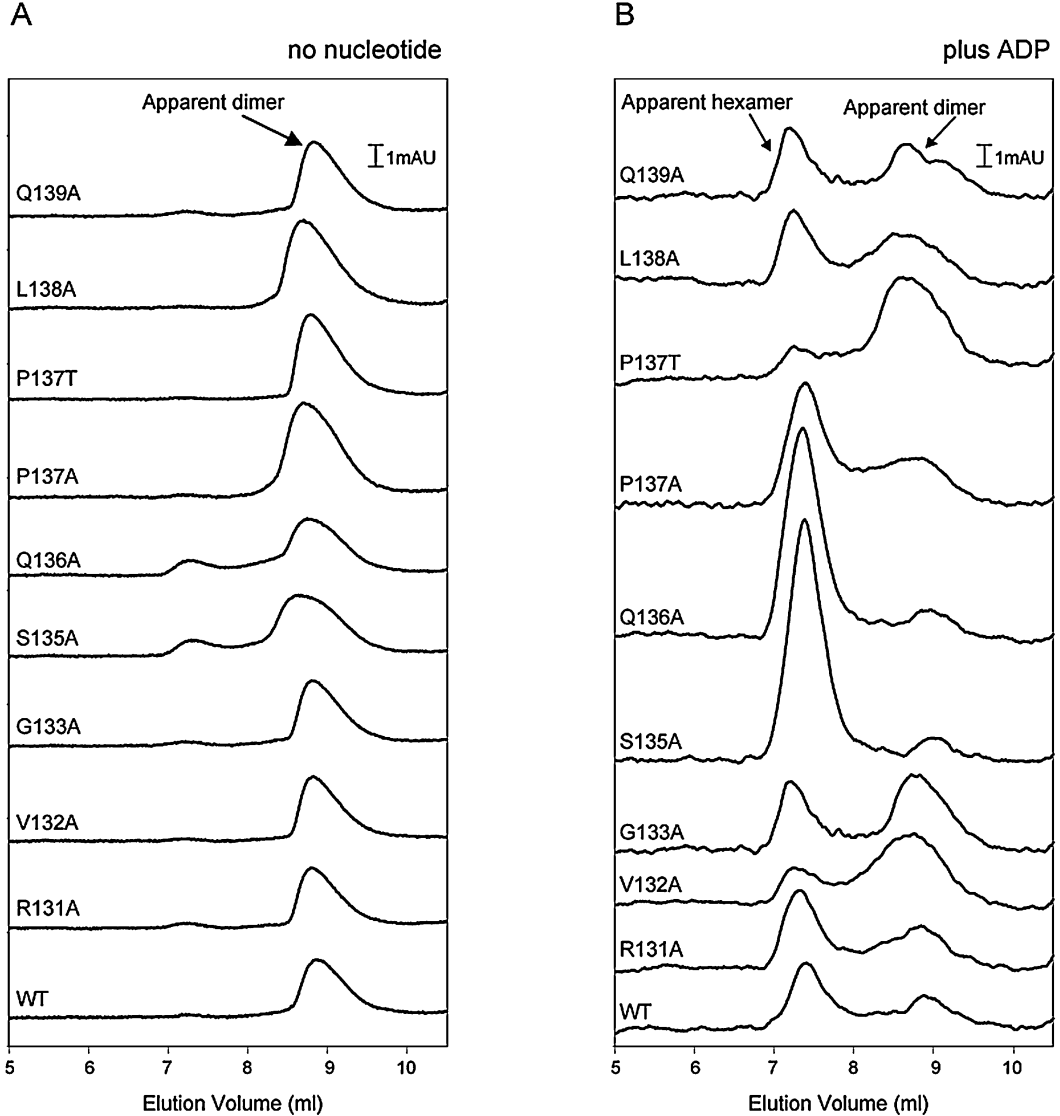
Gel filtration profiles of PspF_1−275_ (WT and variants) in the absence and presence of ADP. Nucleotide-dependent apparent dimer/hexamer equilibrium of PspF_1−275_ proteins as obtained by analytical HPLC gel-filtration chromatography using a Biosep 3000 column (Phenomenex), in the absence (A) and presence (B) of ADP. Chromatographs are overlaid and offset but not normalized. The PspF_1−275_ variants are as indicated.

To determine whether the reduced ATP hydrolysis rates observed in the pre-SIi variants are due to defects in hexamer formation, a prerequisite for maximal hydrolysis rates (Schumacher *et al*., 2004), we performed gel-filtration experiments in the absence and presence of ADP. PspF_1−275_WT normally exists in equilibrium between a dimer and a hexamer at low protein concentrations (below 10 μM), but in the presence of either ATP or ADP the equilibrium shifts to the hexameric species (Joly *et al*., 2006). The pre-SIi variants eluted predominantly as dimers (Fig. 4A) in the absence of nucleotide and hexamers in the presence of ADP (Fig. 4B), although we note that the apparent dimer/hexamer ratios differed from PspF_1−275_WT, especially the V132A, S135A, Q136A and P137T variants. In the case of the S135A and Q136A variants a significant shift towards the hexameric species was observed, whereas the V132A and P137T variants demonstrate a significant defect in their ability to form hexamers, which may account for the reduced ATPase activity of PspF_1−275_V132A. However, we note that PspF_1−275_P137T exhibits PspF_1−275_WT activities in terms of transcription activation and σ^54^ binding. As none of the pre-SIi variants failed to form the apparent hexameric species in the presence of ADP, we suggest that the lack of ATPase activity for the majority of pre-SIi variants is not simply due to the inability to form a hexamer or bind nucleotide.

### The pre-SIi loop stabilizes distinct interactions PspF_1−275_ makes with σ^54^

To ascertain the contribution the pre-SIi loop makes to PspF_1−275_ energy coupling activities, nucleotide-dependent interactions between PspF_1−275_, σ^54^ and/or Eσ^54^ promoter complexes were studied using the protein–DNA cross-linking assay and different ATP analogues: (i) the transition state analogue ADP–AlF; (ii) the ground state analogues AMP–AlF and ADP–BeF; and (iii) the slowly hydrolysable ATP analogue ATPγS.

**Fig. 5:**
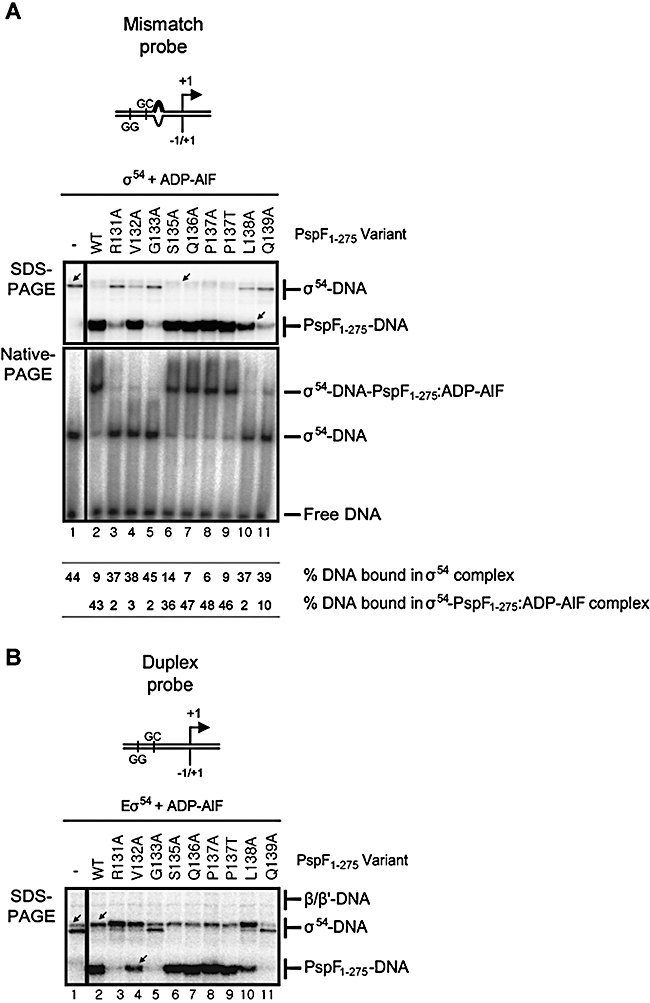
The activities of the V132A and L138A variants are rescued by ADP–AlF. A. SDS-PAGE gel showing the cross-linking profiles of σ^54^–DNA complexes formed on the mismatch promoter probe in the presence of ADP–AlF. The migration positions of the cross-linked σ^54^–DNA and PspF_1−275_–DNA species are indicated. A cross-linked PspF_1−275_–DNA species is observed in reactions containing the V132A (lane 4), S135A (lane 6), Q136A (lane 7), P137A/T (lanes 8–9) and L138A (lane 10) variants. Native-PAGE gel illustrating that stable ADP–AlF trapped complexes are only observed in the presence of PspF_1−275_ WT (lane 2) and the S135A (lane 6), Q136A (lane 7) and P137A/T (lanes 8–9) variants. The migration positions of the σ^54^–DNA-PspF_1−275_:ADP–AlF (trapped) and binary σ^54^–DNA (σ^54^–DNA) complexes, free DNA and percentage DNA bound in each complex are as indicated. B. SDS-PAGE gel as in A but on the duplex promoter probe in the presence of core RNAP. The migration positions of the cross-linked β/β′–DNA, σ^54^–DNA and PspF_1−275_–DNA species are indicated.

The ability of the PspF_1−275_ pre-SIi variants to support stable (trapped) complex formation with σ^54^ and/or Eσ^54^ was first assayed in the presence of the ATP transition state analogue, ADP–AlF (Fig. 5). As illustrated in Fig. 5A (Native-PAGE), only in the presence of the pre-SIi variants S135A, Q136A and P137A/T were ADP–AlF-dependent trapped complexes formed at a similar level to PspF_1−275_WT. When these reactions were analysed by photo-cross-linking (Fig. 5A; SDS-PAGE), we observed a σ^54^-dependent PspF_1−275_–DNA cross-link (PspF_1−275_–DNA; Fig. 5A, lanes 6–9 and Burrows *et al*., 2009). However, we also observed a cross-linked PspF_1−275_–DNA species in reactions containing the V132A (lane 4 arrowed) and L138A variants (lane 10). These data demonstrate that in the presence of ADP–AlF, the effect of the V132A and L138A substitutions are less apparent than in the transcription assays (Fig. 2), although the stability of ADP–AlF-dependent complexes are greatly reduced (as judged by Native-PAGE analysis; Fig. 5A). Similar to the reactions containing PspF_1−275_WT, the V132A (lane 4), S135A (lane 6), Q136A (lane 7), P137A/T (lanes 8–9) and L138A (lane 10) variants caused a change in the σ^54^–DNA profile (Fig. 5A; from a single lane 1 arrowed, to no band lane 6 arrowed). We also observed very weak PspF_1−275_–DNA interactions with the R131A (lane 3), G133A (lane 5) and Q139A (lane 11) variants, although no alteration in the σ^54^–DNA profile was evident. Interestingly, when core RNAP was added to these reactions (Fig. 5B) the very weak PspF_1−275_–DNA interactions with the R131A, G133A and Q139A variants were no longer evident (compare Fig. 5A and B, lanes 3, 5 and 11 respectively); however, we do note a change in the σ^54^–DNA profile (from a double band to a single band) in the reaction containing the R131A variant (lane 3 arrowed), similar to that observed for PspF_1−275_WT (lane 2 arrowed) and the V132A, S135A, Q136A, P137A/T and L138A variants (lanes 4 and 6–10).

In the presence of the ATP ground state analogues AMP–AlF (Joly *et al*., 2008) and ADP–BeF (Burrows *et al*., 2009), similar to the ADP–AlF reactions, only the S135A, Q136A and P137A/T variants supported stable trapped complex formation (Fig. S2A, Native-PAGE). Importantly, only these variants (S135A, Q136A and P137A/T) supported PspF_1−275_–DNA interactions (especially in the presence of core RNAP) and an alteration in the σ^54^–DNA cross-linking profile (from a single band, lane 1 arrowed, to no band, lane 6 arrowed). For the remainder of the pre-SIi variants, no cross-linked PspF_1−275_–DNA species was detected and neither was a change in the σ^54^–DNA profile. In the presence of the slowly hydrolysable ATP analogue, ATPγS, we observed a PspF_1−275_–DNA cross-linked species and an altered σ^54^–DNA profile with the S135A, Q136A and P137A/T variants (Fig. S3). The S135A variant displays a significantly reduced level of PspF_1−275_–DNA cross-linked species, which we attribute to the lower ATPase activity of S135A.

Taken together these data indicate that residues V132 and L138 are organized differently with the ATP hydrolysable (dATP and ATPγS) and ATP ground state (AMP–AlF and ADP–BeF) analogues, than with the ATP transition state analogue (ADP–AlF).

### Evolutionary covariance of the E81, E97 and R131 residues support a conserved switch between the pre-SIi and L1 loops in bEBPs

**Fig. 6:**
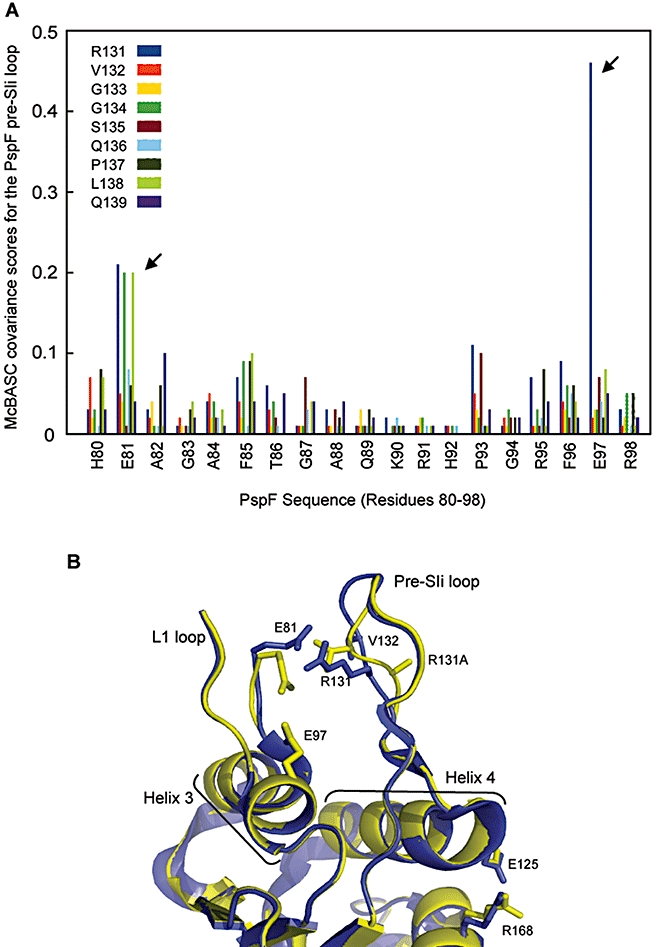
A conserved switch between the pre-SIi and L1 loops exists within bEBPs. A. The covariance between the PspF pre-SIi sequence (RVGGSQPLQ; colour-coded as shown) and PspF residues 80–98 was calculated and depicted graphically. The strong covariance of residue E81 with pre-SIi residues R131, G134 and L138 (arrowed) and residue E97 and pre-SIi residue R131 (arrowed) is indicated by high covariance scores. B. The crystal structures of the apo-PspF_1−275_R131A (yellow) and apo-PspF_1−275_WT (blue; PDB 2BJW) demonstrate the effect of the R131A mutation on the pre-SIi loop conformation. The two structures were aligned on the main chain atoms of residues 35–42. The positions of residues E81 (L1 loop), E125 (Helix 4), R168 (putative R-finger), R131 and V132 (pre-SIi) and the R131A mutation (in the context of the PspF_1−275_R131A structure) are indicated. Structural features relevant to bEBPs such as the L1 and pre-SIi loops, Helix 3 and Helix 4 are labelled. Clear local differences between the apo-PspF_1−275_WT and apo-PspF_1−275_R131A structures are apparent in the pre-SIi loop conformation, as well as a significant rotation of residue E81 (L1 loop) resulting in the disruption of the E81-R131 link.

Our structural and functional data suggest that (i) pre-SIi residues facing the L1 loop are probably required to position the L1 loop for engagement with σ^54^ at distinct steps during the ATP hydrolysis cycle and (ii) some pre-SIi residues, despite having no obvious contact features outside the pre-SIi loop, impact on the ATP hydrolysis and σ^54^ engagement activities of PspF. As the pre-SIi loops of bEBPs demonstrate a degree of sequence conservation (Fig. 1B), this sequence is well suited for predictive covariance analysis as an independent means to identify interacting co-evolved residues. As bEBPs function in a conserved manner, it seems reasonable to suggest that evolutionary variations are susceptible to selective pressures to maintain functionally linked residues. To explore covariation we first aligned the 289 seeded non-redundant sequences of the AAA+ domains of bEBPs (Pfam 00158) using the ClustalW program. Using three established covariance algorithms (Fodor and Aldrich, 2004) [observed minus expected squares, statistical coupling analysis and McLachlan-based substitution correlation (Olmea *et al*., 1999)] we determined the covariance of residues within the conserved pre-SIi sequence and the entire AAA+ domain of bEBPs (Fig. S4). We also determined the covariance between PspF residues 80–98 (which includes the L1 loop sequence) and the PspF pre-SIi loop sequence (Fig. 6A). As shown in Fig. 6A PspF residue E97 (arrowed) exhibits strong covariance with pre-SIi residue R131 and residue E81 (arrowed) exhibits covariance with the pre-SIi residues R131, G134 and L138, supporting the existence of a functionally conserved mechanism by which bEBPs relate the nucleotide-bound state to pre-SIi loop dynamics.

### Conformational changes are apparent in the crystal structure of PspF_1−275_R131A

The severely reduced ATP hydrolysis rates observed for the majority of the pre-SIi variants suggest the existence of an important structural link coupling the pre-SIi loop to the distant ATP hydrolysis site. We reasoned that mutations in the pre-SIi loop would result in local, and potentially global, conformational changes that could reveal mechanistic details of this structural link. Based on our covariance analysis we determined the crystal structure of apo-PspF_1−275_R131A in order to determine the effect of the R131A mutation on the conformation of the pre-SIi loop compared with apo-PspF_1−275_WT (Fig. 6B and Fig. S5A–C). Clear local differences are evident in the conformation of the pre-SIi loop, as well as a significant rotation in the side-chain of residue E81 of the adjacent L1 loop (Fig. 6B). These observations strongly support the view that residue R131 of the pre-SIi loop communicates with residue E81 of the L1 loop in a nucleotide-dependent manner that appears essential for PspF_1−275_ activity. Disruption of the R131-E81 link (by introduction of the R131A mutation) causes the pre-SIi to rotate, so that the alanine side-chain is now pointing towards the other stem of the pre-SIi loop (away from the L1 loop). More globally, the alanine substitution at residue R131 results in conformational changes in Helix 4 (most notably residue E125) and, somewhat surprisingly, the orientation of residue R168 a putative R finger (as shown by the root mean square deviation plot between PspF_1−275_R131A and PspF_1−275_WT; Fig. S5D). Although these differences are modest, they are the most pronounced changes evident over the entire length of the PspF_1−275_ structure outside the pre-SIi and L1 loops.

## Discussion

We have systematically examined the activities of the pre-SIi sequence in PspF_1−275_, characterized by crystallographic studies as a well-defined loop structure that changes its organization and orientation in the presence of different nucleotides (Rappas *et al*., 2006). In PspF_1−275_ and the related bEBPs NtrC1 and ZraR, a direct interaction between the pre-SIi loop and the L1 loop is evident (Lee *et al*., 2003; Sallai and Tucker, 2005). The importance of specific pre-SIi residues in PspF_1−275_ functionality now establishes that particular regions of the pre-SIi loop are important for engaging and remodelling the Eσ^54^ closed complex in response to the ATP hydrolysis cycle.

### PspF_1−275_ pre-SIi integrity is required for high ATP hydrolysis rates

The majority of the pre-SIi variants showed no marked defects in self-association, although we do note that the V132A and P137T variants exhibited lower levels of hexamer formation (which may account for the low ATPase activity of V132A) and the S135A and Q136A variants showed a propensity for the hexameric state (even though the ATPase activity of S135A is severely reduced). Taken together these results suggest that the pre-SIi loop does not contribute greatly to the energetically favourable interactions (at the protomer–protomer interface) needed for hexamer formation. Clearly, alanine substitutions in the pre-SIi loop impact on the catalytic site architecture, reflected by the decrease in ATP hydrolysis rates. Differences in K_M_, notably for the V132A and S135A variants probably reflect differences in hydrolysis rates rather than in ATP binding due to the reported high off-rates (Rombel *et al*., 1999; Schumacher *et al*., 2004). The similar apparent nucleotide binding affinities of all the pre-SIi variants tested (Fig. S1) are consistent with the spatial separation of residues involved in nucleotide binding and hydrolysis (Rappas *et al*., 2005). Apart from the P137A/T variants, all the pre-SIi variants showed drastically reduced ATP hydrolysis rates, which is notable because the pre-SIi is a flexible surface-exposed loop that is spatially distant from the ATP hydrolysis site, suggesting that linked dynamic modes underpin the nucleotide-dependent functions of PspF (Rappas *et al*., 2006).

The crystal structure of apo-PspF_1−275_R131A illustrates that destabilization of the pre-SIi loop–L1 loop interaction results in a significant conformational change in the pre-SIi loop that propagates to the C-terminal moiety of Helix 4 (Fig. 6B). This change appears to weaken the intrasubunit E125–R168 interaction and the orientation of the putative R-finger residue R168. Previously we showed that the R168A variant forms nucleotide-independent hexamers that are ATPase defective (Schumacher *et al*., 2004). These prior observations suggested that residue R168 inhibits hexamer formation in apo-PspF_1−275_WT while being required for intersubunit catalysis in the ATP-bound state (Schumacher *et al*., 2004). Consistent with these findings, we propose that the structural conformation of the pre-SIi loop of apo-PspF_1−275_WT, via Helix 4 residue E125, supports a conformation of R168 that inhibits self-association.

### Pre-SIi residues support different steps during transcription activation

Using different ATP analogues three broad classes of activities associated with the pre-SIi loop were identified. Class I contained variant forms of the pre-SIi loop that had activities that were indistinguishable from PspF_1−275_WT, indicating that these pre-SIi residues (Q136 and P137) are largely unimportant for nucleotide-dependent engagement of the Eσ^54^ closed complex. We also assign S135 to class I because it behaves similarly to PspF_1−275_WT in the nucleotide-dependent interaction studies and the significantly reduced transcription activation and σ^54^ isomerization activities could be attributable to its low ATPase activity. These residues are located farthest away from the L1 loop (Fig. 1A) and are therefore unlikely to interact with the L1 loop. Class II variants (G133, Q139 and to some extent R131) essentially failed in all the nucleotide-dependent interaction assays tested. The severe functional defects observed for PspF_1−275_G133A are not surprising given its high sequence conservation and high φ–ψ angle indicating that backbone flexibility at the tip of the pre-SIi loop appears indispensable for bEBP activity. Crystal structures of PspF_1−275_ illustrate that nucleotide binding causes the pre-SIi loop to twist around its longitudinal axis (Rappas *et al*., 2006). Diminishing the glycine-associated flexibility (in the G133A variant) strongly suggests that the potential for ‘structural twist’ is critically important for PspF functionality. Class II variants maintain the nucleotide binding and self-association activities but cannot engage the Eσ^54^ closed complex, presumably because the contact features for binding σ^54^ (i.e. the L1 loop) are inappropriately presented. Class III variants (represented by V132A and L138A) supported PspF_1−275_–DNA cross-links in the presence of the ATP transition state analogue (ADP–AlF) but not with the ATP ground state analogues (AMP–AlF and ADP–BeF) or the slowly hydrolysable ATP analogue (ATPγS), suggesting that the pre-SIi loop plays at least two distinct roles in productive Eσ^54^–PspF interactions that are dependent on the nucleotide state: weak interactions that exist in the nucleotide ground state and strong interactions in the ATP transition state.

The high probability of pair-wise co-evolution of the L1 residues E81 and E97 (in PspF) with the pre-SIi residue R131 (in PspF) suggests that functionally important contacts between the pre-SIi and L1 loops exist which may provide a mechanistic rational for how different nucleotide states could orientate the L1 loop for Eσ^54^ engagement. R131 would function as switch which pivots between E81 in the ATP ground state and E97 in the ATP transition state. Disruption of this switch may account for the failure of PspF_1−275_R131A to stably interact with σ^54^, as demonstrated by the lack of σ^54^-dependent PspF_1−275_–DNA cross-links with the R131A variant in the presence of the metal fluoride analogues (Fig. 5 and Figs S2 and 3, lane 3). The covariance of residues E81 (L1 loop) and R131, G134 and Q136 (pre-SIi) indicates that additional residues are required for maintaining a pre-SIi loop conformation suitable for a productive E81–R131 interaction to occur (Fig. 6A). Overall, the functionally important R131-E81/E97 switch (studied in PspF) appears highly conserved within the bEBP family.

### The pre-SIi loop does not appear to contact promoter DNA

The DNA-bound model of the electron microscopy structure of Eσ^54^-PspF_1−275_:ADP–AlF indicates that the pre-SIi loop lies proximal to both σ^54^ and promoter DNA (Bose *et al*., 2008). One important observation from the nucleotide-dependent studies is that in the presence of different DNA templates (Fig. 5 and Figs S2 and 3) no large scale changes in the amounts of complexes formed (in Native-PAGE analysis compared with PspF_1−275_WT) were observed, nor was the stability of these complexes increased or reversed, inferring that the pre-SIi loop does not directly contact promoter DNA and/or σ^54^. In contrast, interactions between σ^54^ and the L1 loop variant PspF_1−275_T86S can be recovered when promoter DNA sequences downstream of the −12 position are missing (Dago *et al*., 2007). We propose that much of the action of the pre-SIi loop is manifest through the L1 loop interacting with σ^54^ and that the pre-SIi loop in PspF and other related bEBPs works mainly on the L1 loop rather than its substrate.

### Comparison with the pre-SIi sequence of other AAA+ proteins

The similarity in the location of the synapomorphic pre-SIi feature, which is found in or at the edge of the inner pore of the hexameric AAA+ ring assembly (Iyer *et al*., 2004), raises an important question regarding a common role for the pre-SIi in other AAA+ proteins. In RuvB, which contacts RuvA via the pre-SIi β-hairpin, mutations in the pre-SIi result in decreased RuvB ATPase activity (Han *et al*., 2001; Yamada *et al*., 2002), while in E1 helicase of Papilloma virus, the pre-SIi β-hairpin interacts with single-stranded DNA (Enemark and Joshua-Tor, 2006). In these cases the pre-SIi contacts auxiliary proteins or substrates directly, for which we found no evidence in PspF. In MCM, a pre-SIi mutation modestly increased the *K*_d_ for DNA binding, but significantly reduced ATPase and abolished helicase activity (McGeoch *et al*., 2005), suggesting tight structural coupling between the pre-SIi and the hydrolysis site, similar to our observations with PspF. Distinct functional roles that depend on the nucleotide-bound state have, as far as we know, not been shown for other AAA+ pre-SIi sequences. However, structural studies of the E1 helicase (Enemark and Joshua-Tor, 2006) and the E1-related Ltag helicase (Gai *et al*., 2004) suggest that different nucleotide states result in distinct orientations of the pre-SIi. More recently, nucleotide state-dependent dynamics for the pre-SIi have also been proposed for MCM (Barry *et al*., 2009) and ClpX (Martin *et al*., 2008a,b;).

Clearly, the divergence in pre-SIi sequences among members of the pre-SIi β-hairpin superclade of AAA+ proteins and their distinct direct or indirect involvements in substrate remodelling do not allow the simple assignment of common roles to this structural feature. Rather, the strategic placement of the pre-SIi near or in the pore of the AAA+ ring assembly, and the potential structural coupling to the ATP hydrolysis event may explain the marked evolutionary prevalence of the pre-SIi.

## Experimental procedures

### Proteins and DNA probes

*Escherichia coli* core RNAP was purchased from Epicentre technology (Cambio). *Klebsiella pneumoniae*σ^54^ was purified as described (Wigneshweraraj *et al*., 2003). Plasmid pPB1 encoding *Escherichia coli* PspF residues 1–275 (PspF_1−275_ with an N-terminal His6 tag in pET28b+) was mutagenized (Quickchange Mutagenesis Kit, Stratagene) resulting in plasmids pPB1(R131A), pPB1(V132A), pPB1(G133A), pPB1(G134A), pPB1(S135A), pPB1(Q136A), pPB1(P137A), pPB1(P137T), pPB1(L138A) and pPB1(Q139A). Proteins were overproduced as described (Wigneshweraraj *et al*., 2003). Proteins were purified using a gravity flow method. Briefly Ni-NTA resin (Qiagen) was equilibrated with buffer A_NI_ (20 mM sodium phosphate pH 7.0, 500 mM NaCl, 5% (v/v) glycerol) prior to applying the cell supernatant. The resin was then washed with buffer A_NI_, followed by wash buffer 1 (buffer A_NI_ plus 40 mM imidazole) and wash buffer 2 (buffer A_NI_ plus 80 mM imidazole). The protein was eluted using buffer B_NI_ (buffer A_NI_ plus 1 M imidazole). The *S. meliloti nifH* phosphorothioated (Operon) DNA probes were derivatized with *p*-azidophenacyl bromide as described (Burrows *et al*., 2004). The modified promoter strands were ^32^P labelled and annealed to the complementary strand as described (Wigneshweraraj *et al*., 2003; Burrows *et al*., 2008).

### In vitro full-length or abortive transcription assays

Full-length or abortive transcription assays were performed in STA buffer (25 mM Tris-acetate pH 8.0, 8 mM Mg-acetate, 10 mM KCl, 3.5% w/v PEG 6000) in a 10 μl reaction containing 100 nM Eσ^54^ (reconstituted at a 1:4 ratio of E:σ^54^), 4 mM dATP and 20 nM promoter DNA probe. The mix was incubated at 37°C for 5 min and the reaction started by addition of 5 μM PspF_1−275_WT or variants and incubated for a further 10 min at 37°C. Full-length transcription was initiated by adding an elongation mix containing 100 μg ml^−1^ heparin, 1 mM ATP, CTP, GTP, 0.05 mM UTP and 3 μCi [α-^32^P]-UTP and incubated at 37°C for 20 min. The full-length transcription products were analysed on a 6% denaturing gel. Synthesis of the abortive transcript (5′ UpGGG) was initiated by adding an abortive mix containing 100 μg ml^−1^ heparin, 0.5 mM 5′ UpG and 4 μCi [α-^32^P]-GTP and incubated at 37°C for 20 min. The abortive transcription products were analysed on a 20% denaturing gel. Full-length and abortive transcription gels were visualized and quantified using a Fuji FLA-5000 PhosphorImager.

### ATP binding and hydrolysis

ATP binding assays of PspF_1−275_ proteins by UV cross-linking were performed as described (Schumacher *et al*., 2004). ATPase reactions were carried out as described (Schumacher *et al*., 2008). ATPase assays were performed in reaction buffer (35 mM Tris-acetate pH 8.0, 70 mM K-acetate, 5 mM Mg-acetate, 19 mM ammonium acetate, 0.7 mM DTT) containing 15 mM MgCl_2_ and incubated at 23°C, using 0.06 μCi [α-^32^P]ATP or [α-^32^P]-dATP as nucleotide tracers. Prior to ATP titration experiments, protein concentration titrations were carried out at 0.1 mM ATP to verify maximal turnover rates were achieved. Reactions were quenched with 5 vols of 2 M formic acid. [α-^32^P]-ADP was separated from [α-^32^P]-ATP by thin-layer chromatography [on Polygram Cel 300 PE (polyethyleneimine)] and visualized and quantified using a Fuji FLA-5000 PhosphorImager. ATP titration experiments and all PspF_1−275_ titrations were designed so that only 10–30% of the initial amount of ATP was hydrolysed thereby ensuring that any ADP produced had no measurable effect on ATP hydrolysis rates. It also ensures that ATP was not limiting in the assays and that non-linearity of turnover rates was minimal. *V*_max_ and *K*_m_ values as well as standard errors were determined at initial concentrations of 0.05–1 mM ATP using non-linear regression Graphit software (Erithacus Software Limited).

### Gel filtration chromatography

Gel filtration chromatography was carried out with 50 μM PspF_1−275_ (WT and variants) at room temperature in running buffer (20 mM Tris pH 8.0, 50 mM NaCl, 15 mM MgCl_2_, 0.02% (w/v) azide) at a 0.7 ml min^−1^ flow rate using a BioCad Sprint HPLC system and a Bio-Sep-S 3000 column (Phenomenex). To test for nucleotide-dependent oligomerization the column was pre-equilibrated with five bed volumes of 0.5 mM ADP, prior to loading 10 μM PspF_1−275_ (WT or variants), supplemented with 0.5 mM ADP. The column was calibrated using molecular weight standards (Sigma): Blue Dextran (200 kDa), Thyroglobulin (669 kDa), Ferritin (440 kDa), Catalase (232 kDa), Aldolase (158 kDa), Albumin (67 kDa), Ovalbumin (43 kDa), Chymotrypsin (25 kDa) and Ribonuclease (13.5 kDa).

### Photo-cross-linking assays

Cross-linking reactions were conducted at 37°C in STA buffer in a 10 μl reaction volume as described (Burrows *et al*., 2008; 2009). Briefly, where indicated either 1 μM σ^54^ or 200 nM Eσ^54^ (reconstituted using a 1:2 ratio of E:σ^54^) and 20 nM modified ^32^P-labelled promoter DNA probe was incubated for 5 min at 37°C. Open complexes were formed by 4 mM dATP and 5 μM PspF_1−275_ and the reaction incubated for 10 min at 37°C. Trapped complexes were formed *in situ* by adding 5 μM PspF_1−275_, 5 mM NaF and either 1 mM ADP, 0.2 mM BeCl_3_ for the ADP–BeF reactions; 1 mM ADP, 0.2 mM AlCl_3_ for the ADP–AlF reactions; and 1 mM AMP, 0.2 mM AlCl_3_ for the AMP–AlF reactions and incubated for 10 min at 37°C. To eliminate free core RNAP from binding the promoter probe, reactions contained 100 ng ml^−1^ salmon sperm DNA. Reactions were UV irradiated at 365 nm for 30 s using a UV-Stratalinker 1800 (Stratagene). A 2 μl sample of the reaction was analysed by Native-PAGE (4.5%), run at 100 V for 55 min. The remainder of the cross-linking reaction was diluted using 5 μl 10 M Urea and 5 μl 2× SDS loading buffer (Sigma), heated at 95°C for 3 min and 10 μl loaded on a 7.5% SDS-PAGE gel run at 200 V for 50 min. The gels were visualized and quantified using a FLA-500 PhosphorImager. The cross-linked proteins were identified using antibodies as described (Burrows *et al*., 2008).

### Covariance analysis

Covariance analysis was performed as described (Fodor and Aldrich, 2004). Briefly, the 289 sequences in Pfam (00158) were first aligned using ClustalW. For a multiple alignment of N sequences, an NxN matrix (with dimensions k and l) was constructed for each pair of residues (i and j). Each entry in the matrix was then scored using the McLachlan substitution matrix (Olmea *et al*., 1999), producing high scores for identities/conservative substitutions and low scores for non-conservative substitutions. The correlation between the two columns (*i* and *j*) was calculated using the equation 
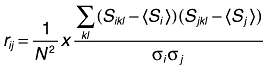
 where <*S*> is the average and *σ*_*i*_ is the standard deviation of all entries in the matrix.

### Crystallization and structure determination

PspF_1−275_R131A was expressed, purified, crystallized and flash frozen as described (Rappas *et al*., 2005). Diffraction data were collected (in-house) using a March 345 image plate mounted on a Rigaku RU-H3RHB rotating-anode X-ray generator (operated at 50 kV and 100 mA) fitted with Osmic confocal optics and a copper target (Cu *K*α; λ= 1.542 Å). All the data sets were processed using MOSFLM (Leslie, 1992) and SCALA (Evans, 2006) using the CCP4 program suite (Collaborative Computational Project, 1994). Phases for PspF_1−275_R131A crystals were obtained by molecular replacement using the PspF_1−275_WT (PDB 2BJW) structure as a search model and the PHASER program (Storoni *et al*., 2004). Flexible regions such as the L1 (residues 79–93) and pre-SIi loops were first removed from the search model and built-in during rounds of refinement. The F_o_-F_c_ and 2F_o_-F_c_ maps showed some connected electron density for residues 79–81, 91–93 and 129–135, which were manually built-in using program O (Jones *et al*., 1991). After several rounds of rebuilding, minimization and *B*-individual refinements, water molecules were added in the F_o_-F_c_ map automatically (using CNS) and manually. A final cycle of TLS refinement was carried out using the REFMAC5 program (Murshudov, 1997) and the two TLS groups: the α/β subdomain (residues 7–177) and the α-helical domain (residues 180–255). The refinement statistics are summarized in Table S1. All figures were prepared using Pymol (DeLano, W.L. The PyMOL Molecular Graphics System 2002).
