# Supplementary material for: Functional roles of the pre-sensor I insertion sequence in an AAA+ bacterial enhancer binding protein
Source: Mol Microbiol. 2009 Jun 23;73(4):519–33. doi: 10.1111/j.1365-2958.2009.06744.x (PMC2745333; doi:10.1111/j.1365-2958.2009.06744.x)

Supplementary Information

Supplementary Table 1: Crystallographic data and refinement statistics of the PspF$_{1\text{-}275}$R131A structure

Space group	P6$_5$
Unit Cell (Å)	a = b = 113.8, c = 39.4<br>α = β = 90º, γ = 120º

A. *Data reduction statistics*

λ (Å)	Resolution (Å)	Unique reflections	Redundancy	I / σ	Completeness (%)	Rsym[a] (%)
1.54179	32.86 – 2.21 (2.33 – 2.21)	14918	9.7	29.9 (8.2)	99.9	6.2 (25.8)

B. *Refinement Statistics*

Reflections (work/free)	14134/767
Number of atoms	2050 (135 water molecules)
R_{work} (%)	17.3
R_{free} (%)	23.5
**Ramachandran plot (%)**	
Favoured	95.7
Generous	0.5
Disallowed	0
**rms deviation from real values**	
Bond Lengths (Å)	0.019
Bond angles (deg.)	1.7

[a]Rsym= Σi/Ii - <*I*>|Σ<*I*>, where Ii and <*I*> are the observed and averaged intensities.

The numbers in brackets are for the highest resolution shell.

Supplementary Figure Legends

Supplementary Fig. 1: Nucleotide binding using UV-crosslinking of [α-^{32}P]ATP to PspF$_{1\text{-}275}$ proteins.

Following UV irradiation of 20 μM PspF$_{1\text{-}275}$ proteins in the presence of 40 μCi [α-^{32}P] , and SDS-PAGE analysis, PspF$_{1\text{-}275}$ bands of equal Coomassie-blue intensities containing covalently cross-linked radioactivity were quantified by phosphorImager analysis and expressed in photo-stimulated luminescence. The variants are as indicated. The negative control refers to the Walker B mutation PspF$_{1\text{-}275}$K42A, which is proposed to be monomeric and as such unable to interact with nucleotide (Schumacher *et al.*, 2004). Here "-" represents the absence of protein.

Supplementary Fig. 2: The pre-SIi variants V132A and L138A are defective in the presence of the ATP ground state analogues, AMP-AlF and ADP-BeF. A) SDS-PAGE gel showing the cross-linking profiles of σ^{54}-DNA complexes formed on the mismatch promoter probe in the presence of *in situ* generated AMP-AlF (see Experimental Procedures). The migration positions of the cross-linked σ^{54}-DNA and PspF$_{1\text{-}275}$-DNA species are indicated. Cross-linked PspF$_{1\text{-}275}$-DNA species are no longer observed in reactions containing V132A and L138A. Native-PAGE gel illustrating that AMP-AlF-dependent trapped complexes are only observed in the presence of PspF$_{1\text{-}275}$ WT (lane 2) and the pre-SIi variants S135A (lane 6), Q136A (lane 7) and P137A/T (lanes 8-9). The migration positions of the σ^{54}-DNA-PspF$_{1\text{-}275}$:AMP-AlF (trapped) complex, binary σ^{54}-DNA (σ^{54}-DNA) complex, free DNA and percentage DNA bound in each of

the complexes is indicated. **B)** SDS-PAGE gel as in **A)** but on the duplex promoter probe in the presence of core RNAP. The migration positions of the cross-linked β/β'-DNA, σ^{54}-DNA and $PspF_{1-275}$-DNA species are indicated. **C)** SDS-PAGE gel showing the cross-linking profiles of σ^{54}-DNA complexes formed on the mismatch promoter probe in the presence of *in situ* generated ADP-BeF. The migration positions of the cross-linked σ^{54}-DNA and $PspF_{1-275}$-DNA species are indicated.

Supplementary Fig. 3: The pre-SIi variant S135A is affected by the slowly hydrolysable ATP analogue, ATPγS. A) SDS-PAGE gel showing the cross-linking profiles of σ^{54}-DNA complexes formed on the mismatch promoter probe in the presence of ATPγS. The migration positions of the cross-linked σ^{54}-DNA and $PspF_{1-275}$-DNA species are indicated. Cross-linked $PspF_{1-275}$-DNA species are no longer observed in reactions containing S135A. Native-PAGE gel illustrating that the ATPγS-dependent complex (σ^{54}-DNA-$PspF_{1-275}$:ATPγS) formed is unstable and only presence in reactions containing $PspF_{1-275}$ WT (lane 2) and the pre-SIi variants P137A/T (lanes 8-9). The migration positions of the σ^{54}-DNA-$PspF_{1-275}$:ATPγS complex, binary σ^{54}-DNA (σ^{54}-DNA) complex, free DNA and percentage DNA bound in each of the complexes is indicated. **B)** SDS-PAGE gel as in **A)** but on the duplex promoter probe in the presence of core RNAP. The migration positions of the cross-linked β/β'-DNA, σ^{54}-DNA and $PspF_{1-275}$-DNA species are indicated.

Supplementary Fig. 4: A conserved switch between the pre-SIi and L1 loops exists within bEBPs. The co-variance between the pre-SIi consensus sequence (RVGGNKPIK)

and each of the residues that constitute the AAA+ domain of bEBPs (Pfam 00158) was calculated. The correlation between each of the consensus sequence residues (colour-coded as shown) and each residue of the AAA+ domain is depicted graphically.

Supplementary Fig. 5: Crystal structure of the PspF$_{1-275}$R131A variant. A) Interaction between L1 loop residue E81 and the pre-SIi R131 residue (side chains shown as sticks) is disrupted in the R131A structure and the main chain of R131A rotates away from the L1 loop. Structural features relevant to bEBPs such as the L1 loop, the pre-SIi loop, Helix 3 and Helix 4 and the two sub-domains of the AAA+ domain are indicated. **B)** Final $2F_o$-F_c map of the pre-SIi region displayed at 1σ in blue mesh at 2.21Å. Inside the electron density map is the C_α trace model, with the position of R131A indicated. Water molecules (red spheres) added towards later stages of refinement are also visible. **C)** The pre-SIi loop within a simulated annealing omit F_o-F_c map contoured at 3σ, with residues 129-130 omitted. **D)** Comparison of main-chain RMSD values between PspF$_{1-275}$WT and PspF$_{1-275}$R131A. The peak represents residues 130-132, which in the context of PspF$_{1-275}$R131A undergo the most dramatic change (with respect to PspF$_{1-275}$WT). The missing residues in the graph refer to the non-defined L1 loop residues (82-89), which are absent in the crystal structure due to the flexibility of the L1 loop (also see Fig. 1A).

References:

Schumacher, J., Zhang, X., Jones, S., Bordes, P., and Buck, M. (2004) ATP-dependent transcriptional activation by bacterial PspF AAA+protein. *J Mol Biol* **338**: 863-875.

Supplementary Figure 1 Burrows et al

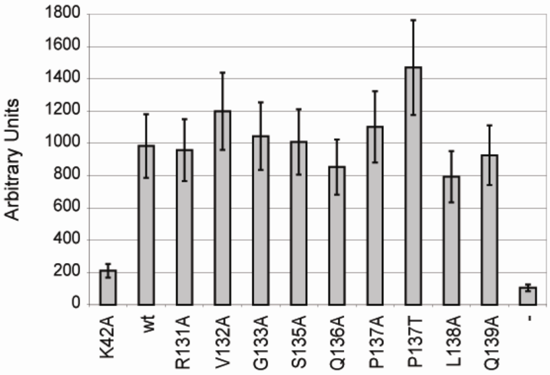

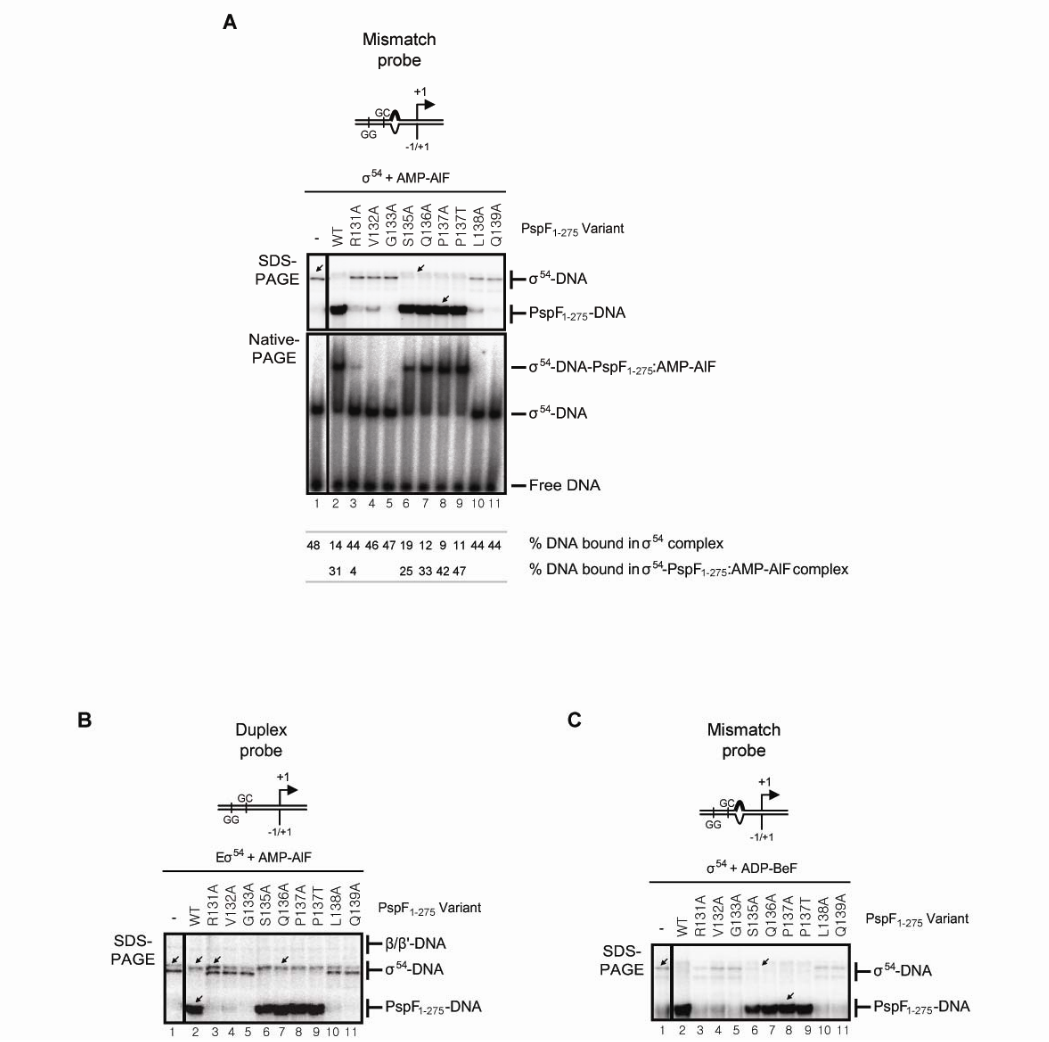
A
Mismatch probe
+1
GC
GG
-1/+1
σ54 + AMP-AlF
PspF1-275 Variant
- WT R131A V132A G133A S135A Q136A P137A P137T L138A Q139A
SDS-PAGE
Native-PAGE
σ54-DNA
PspF1-275-DNA
σ54-DNA-PspF1-275:AMP-AlF
σ54-DNA
Free DNA
1 2 3 4 5 6 7 8 9 10 11
48 14 44 46 47 19 12 9 11 44 44
% DNA bound in σ54 complex
31 4 25 33 42 47
% DNA bound in σ54-PspF1-275:AMP-AlF complex
B
Duplex probe
+1
GC
GG
-1/+1
Eσ54 + AMP-AlF
PspF1-275 Variant
- WT R131A V132A G133A S135A Q136A P137A P137T L138A Q139A
SDS-PAGE
β/β'-DNA
σ54-DNA
PspF1-275-DNA
1 2 3 4 5 6 7 8 9 10 11
C
Mismatch probe
+1
GC
GG
-1/+1
σ54 + ADP-BeF
PspF1-275 Variant
- WT R131A V132A G133A S135A Q136A P137A P137T L138A Q139A
SDS-PAGE
σ54-DNA
PspF1-275-DNA
1 2 3 4 5 6 7 8 9 10 11

Supplementary Figure 3 Burrows et al

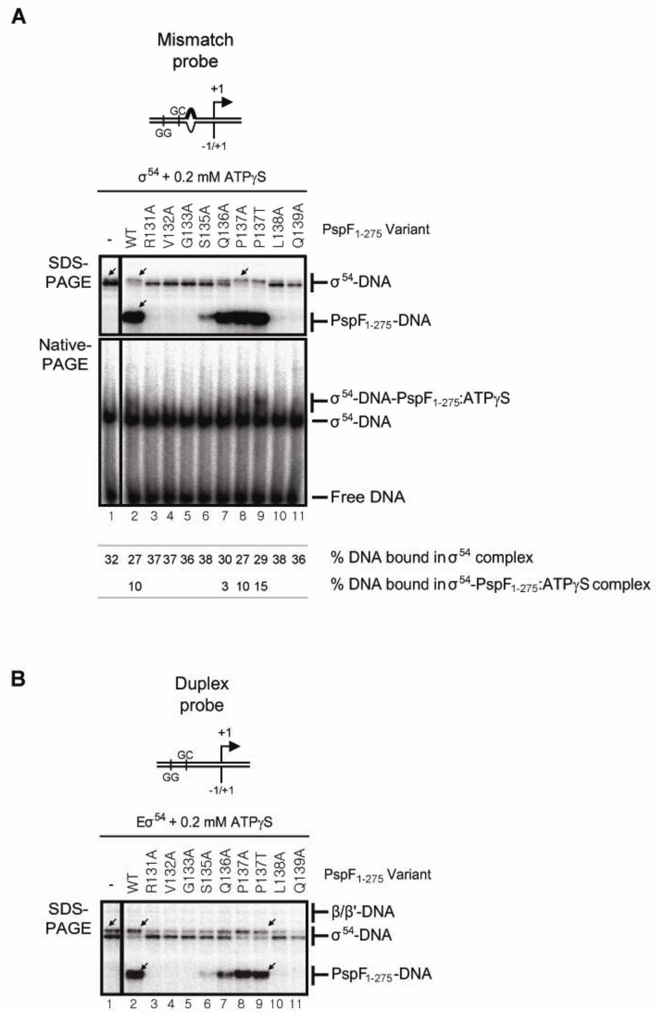

Supplementary Figure 4 Burrows et al

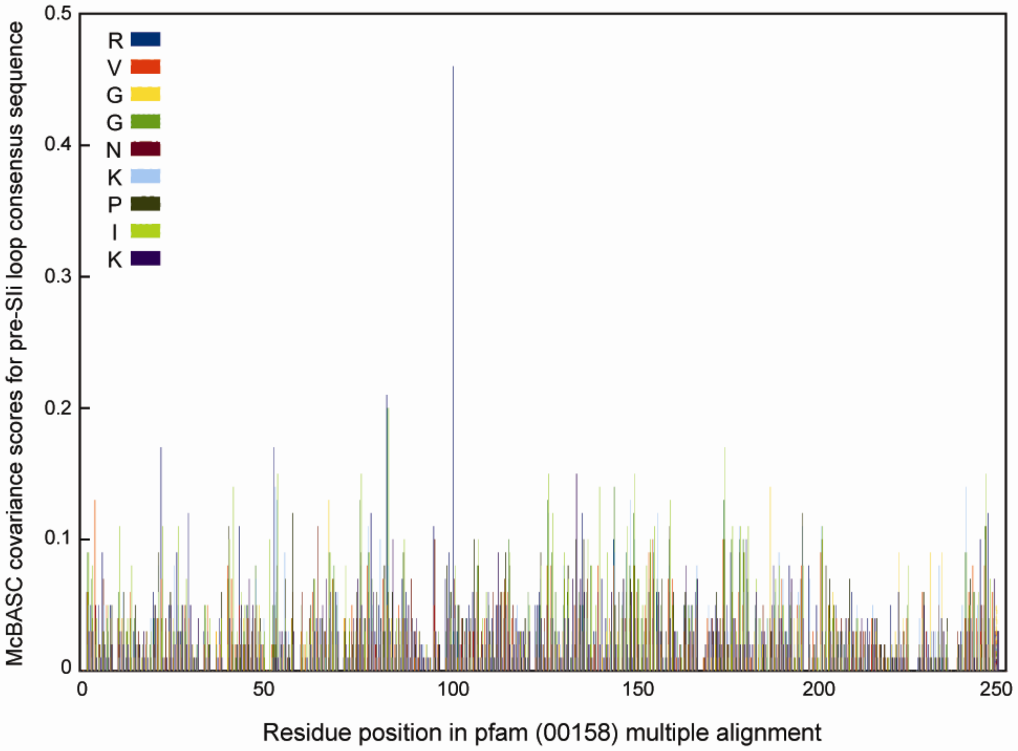

Supplementary Figure 5 Burrows et al

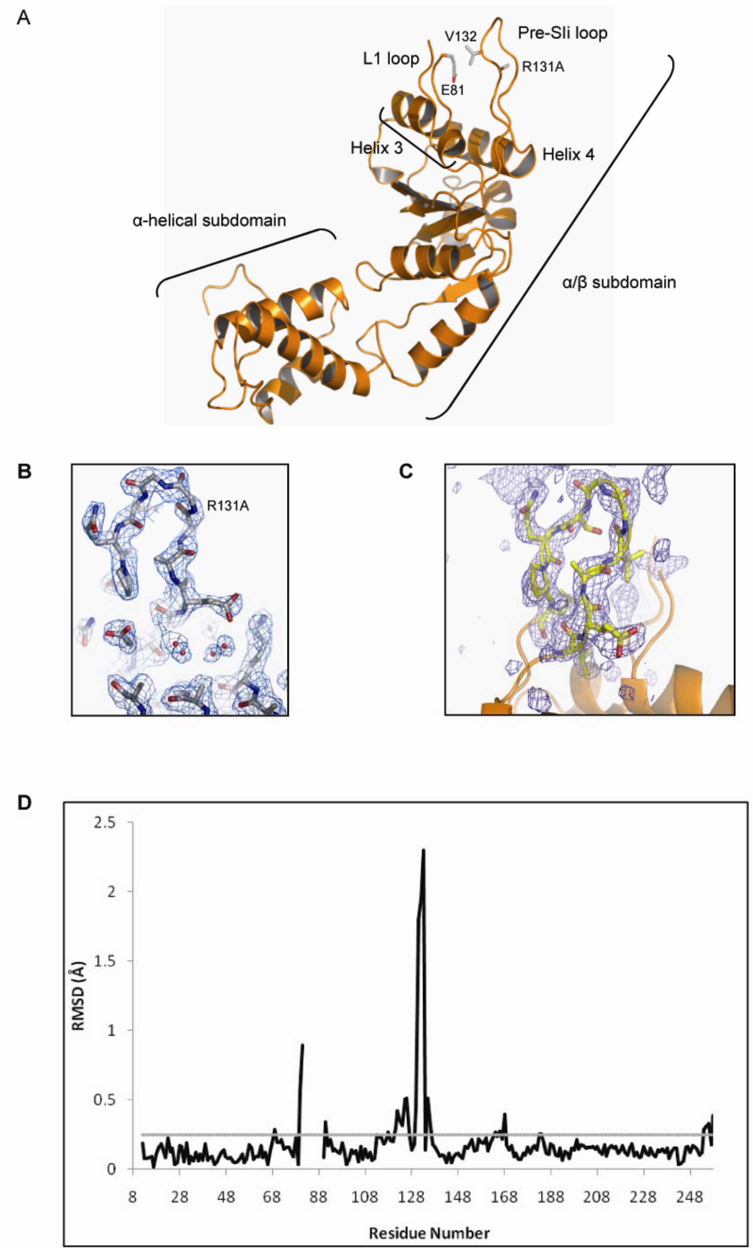

Supplement: Supplementary file 1 [file mmi0073-0519-SD1.pdf]
